# Exploring the Spatial Heterogeneity of Residents' Marginal Willingness to Pay for Clean Air in Shanghai

**DOI:** 10.3389/fpubh.2021.791575

**Published:** 2021-12-24

**Authors:** Ziliang Lai, Xinghua Liu, Wenxiang Li, Ye Li, Guojian Zou, Meiting Tu

**Affiliations:** ^1^The Key Laboratory of Road and Traffic Engineering, Ministry of Education, Shanghai, China; ^2^College of Transportation Engineering, Tongji University, Shanghai, China; ^3^Department of Traffic Engineering, Business School, University of Shanghai for Science and Technology, Shanghai, China; ^4^University of Paris-Saclay, Automatic, School of Information and Communication Technologies, Paris, France

**Keywords:** air pollution, housing prices, geographically weighted regression, marginal willingness to pay, spatial heterogeneity

## Abstract

Previous studies have paid little attention to the spatial heterogeneity of residents' marginal willingness to pay (MWTP) for clean air at a city level. To fill this gap, this study adopts a geographically weighted regression (GWR) model to quantify the spatial heterogeneity of residents' MWTP for clean air in Shanghai. First, Shanghai was divided into 218 census tracts and each tract was the smallest research unit. Then, the impacts of air pollutants and other built environment variables on housing prices were chosen to reflect residents' MWTP and a GWR model was used to analyze the spatial heterogeneity of the MWTP. Finally, the total losses caused by air pollutants in Shanghai were estimated from the perspective of housing market value. Empirical results show that air pollutants have a negative impact on housing prices. Using the marginal rate of transformation between housing prices and air pollutants, the results show Shanghai residents, on average, are willing to pay 50 and 99 Yuan/m^2^ to reduce the mean concentration of PM_2.5_ and NO_2_ by 1 μg/m^3^, respectively. Moreover, residents' MWTP for clean air is higher in the suburbs and lower in the city center. This study can help city policymakers formulate regional air management policies and provide support for the green and sustainable development of the real estate market in China.

## Introduction

In recent years, the continuous increases in energy demand, industrial expansion, and private car ownership in megacities have led to a serious deterioration of air quality (1). According to the 2019 Bulletin on the State of China's Ecology and Environment, in 2019, only 157 of 337 cities at or above the prefectural level met the air quality standard in China, while 180 cities exceeded the standard. Air pollution has serious impacts on residents' health, the development of the regional economy, housing values, and the marginal willingness to pay (MWTP) for clean air (2–6). A recent study pointed out that air pollution in Europe causes an average of 2.2 years of lost life expectancy and about 1.85 million deaths from respiratory diseases each year (7). Similarly, as northern China is dominated by coal-fired heating, the concentration of air pollutants in northern cities will be significantly higher than that in the south, which has caused the average life expectancy of residents in northern areas to decrease by 3.1 years (8).

To achieve a win-win situation between public health and economic development, quantifying the economic value of air quality is very important for the sustainable development of cities. However, previous research on the MWTP for urban clean air has mainly focused on developed countries, such as those in Europe and the United States (9–14). The purpose of this study is to use the latest data to measure residents' MWTP for clean air in different regions of the city, which can improve public health and provide scientific support for the development of region-specific air quality improvement programs and policies.

We choose the impact of air pollutants on housing prices as residents' MWTP for clean air. The MWTP for clean air cannot be directly queried or measured because it is not directly reflected in capital market transactions. However, we can infer the MWTP for clean air indirectly by discussing the relationship between air quality and housing prices (11). As early as the 1960s, some researchers began to explore the complex relationship between air pollutants and housing prices. Ridker and Henning (15) first discovered the negative impact of sulfide on housing prices by using Washington's air quality data in 1967. A few years later, in 1971, Anderson and Crocker also confirmed that air pollutants can reduce people's expectations for the housing market (16). In recent years, Bajari et al. found that housing prices in polluted areas were relatively lower than those in areas with better environmental quality by using the housing transaction data of California from 1990 to 2006 (17). Zheng et al. (18), Zou (19), and Chen and Jin (20) find the same evidence in China.

As one of the factors affecting the quality of life, air quality is considered an environmental factor affecting housing prices, together with other built environmental factors, such as transportation, shopping, entertainment, and education (21, 22). According to the spatial equilibrium model of urban economics, housing prices can reflect residents' MWTP for environmental factors, including clean air (23, 24). Improving air quality will increase the value of houses, and this increase will be passed on to the real estate market in the form of rising housing prices or rents (25–27). In addition, residents' expectations of air quality also affect current housing prices (28). If real estate owners expect the air quality around their houses to rise in the future, they will have higher estimates of current housing prices, and residents will pay more for the expected clean air. Therefore, as the research object of the MWTP for clean air, housing price is a very appropriate choice.

The hedonic method is the most representative and widely used statistical method for revealing the complex relationship between the built environment and housing prices (1, 4, 29). Rosen first adopted the hedonic method to estimate the impact of specific site facilities and clear air on the value of real estate in 1974, and then this method began to be very popular in the research of the impact of the built environment on house prices (30). The hedonic method shows that the quality difference of a commodity is a function of its own attributes, and the gradient difference of price reflects people's MWTP of the commodity (18). Because people do not actively pay for clean air, the hedonic method helps to create a virtual market to measure the hidden value of clean air (11). Therefore, many researchers have used this method to quantify people's MWTP for clean air, as shown in Table 1.

**Table 1:** Research about the impact of air pollution on the real estate market.

**References**	**Study area**	**Method**	**Air pollutant**	**Conclusion**
Smith and Huang (14)	U.S. cities	The hedonic method (MAD and OLS)	TSPs (total suspended particulates)	A decrease in TSPs of 1 μg/m^3^ results in a 0.05–0.10% increase in property values
Zabel and Kiel (11)	U.S. cities	The hedonic method (OLS estimates)	TSPs	The average benefit per household from reducing TSP from 246 to 150 is $137
Chay and Greenstone (9)	U.S. cities	The hedonic method	TSPs	1 μg/m^3^ reduction in TSPs increases the value of housing by 0.2–0.4%
Yusuf and Resosudarmo (10)	Jakarta, Indonesia	The hedonic method (OLS estimates)	THC, SO_2_, and CO	Per family value of clean air in Jakarta ranges from $28 to $85 per μg/m^3^
Le Boennec and Salladarre (13)	Nantes, France	The hedonic method	NOx	Air pollution had no significant impact on the housing price
Chen et al. (1)	Shanghai, China	The hedonic method (OLS estimates)	SO_2_ and PM_10_	The property value would drop by 159 and 238 Yuan/m^2^ when the mean concentrations of SO_2_ and PM_10_ rise by 1 μg/m^3^
Carriazo and Gomez-Mahecha (12)	Bogota, Colombia	The hedonic method	PM_10_	An increase of 1 μg/m^3^ is accompanied by a monthly average rent reduction of 0.61 % for apartments
Zou (19)	282 prefecture-level cities in China	Combing OLS and GWR model	PM_2.5_	A 1 μg/m^3^ increase in the PM_2.5_ is associated with up to a 36 Yuan/m^2^ reduction in housing prices
Chen and Jin (20)	286 prefectural-level cities in China	The econometric model (OLS estimates)	PM_2.5_	A 10% increase in PM_2.5_ concentrations causes a 2.4% reduction in local housing prices
Dong et al. (5)	282 prefecture-level cities in China	Spatial dobbin model	PM_2.5_	A 1 μg/m^3^ increase in the PM_2.5_ is associated with up to a 22.7 Yuan/m^2^ reduction in housing prices

The hedonic method is generally implemented by ordinary least squares (OLS) regression (1, 9, 10, 12, 13). The basic assumption of OLS methods is that the data on housing prices in different regions are spatially independent and static. However, due to the local interaction and spatial instability between different regions, housing prices and their influencing factors show strong spatial heterogeneity, and the OLS method is not applicable because it ignores spatial changes (31). Therefore, some scholars have proposed using spatial econometric models to explore the spatial relationship of house prices (32–35). The geographically weighted regression (GWR) model is a statistical regression model proposed by British scholars (36) to account for the spatial heterogeneity of variables, and it is a kind of spatial econometric model. It is generally believed that GWR models improve upon the traditional spatial regression method, and various studies have shown that GWR models are the best method for exploring the spatial heterogeneity of housing prices and their influencing factors (37–40). Therefore, we use a GWR model to explore the relationship between housing prices and air quality.

To sum up, most of previous studies explored residents' MWTP for clean air from a macro perspective (focusing on major cities in China) (5, 19, 20, 31, 41) and a micro perspective (focusing on communities, considering the building structure, such as the floor area and house age) (1). There is a lack of meso level research, which focuses on each census tract of a city. To fill this gap, this study explores residents' MWTP for clean air from a meso perspective. Specifically, this study makes the following three contributions: First, an empirical study was conducted in Shanghai using a GWR model to explore the spatial heterogeneity of the MWTP for clean air in each census tract. Second, the latest data were used to quantify the economic value of urban clean air and to measure residents' MWTP in different census tracts, which is conducive to achieving a win-win situation between public health and economic development. Third, the actual losses caused by air pollution in Shanghai were computed from the perspective of asset value depreciation in the entirety of the Shanghai housing market. The results of this study can provide a reference for the government to formulate regional air pollution prevention and control policies.

The remainder of this paper is structured as follows. In section Data and Variables, the datasets and initial variables used in this study are described. In section Methodology, the basic framework of our GWR model is introduced. Section Results and Discussion discusses the main results of the empirical study, quantifies the MWTP for clean air, and estimates the total losses caused by air pollution in Shanghai. Finally, section Conclusions and Recommendations provides the main conclusions and recommendations of this study.

## Data and Variables

### Study Area and Background

**Figure 1:**
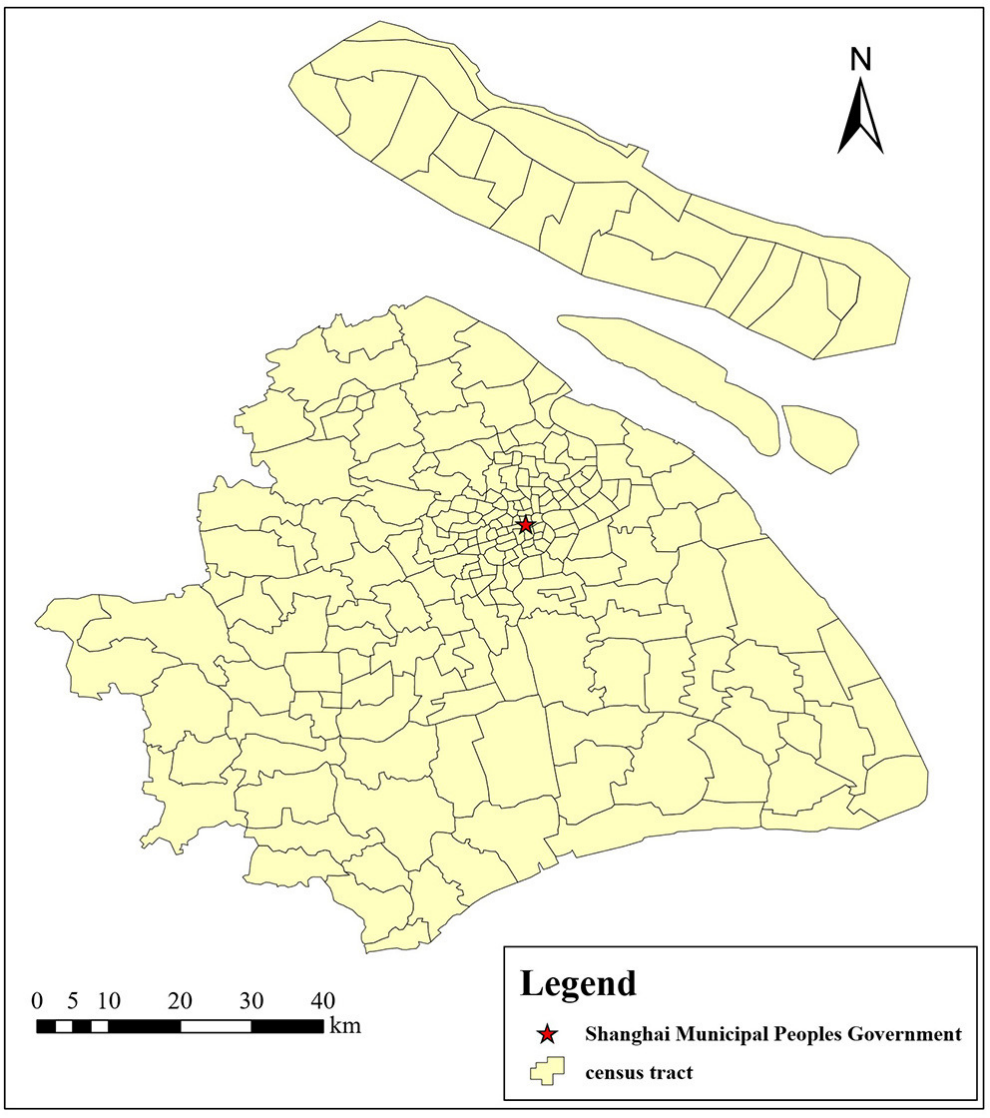
Study area.

Shanghai, the most populous urban area in China, is also China's international economic, financial, trade, shipping, and innovation center. As of 2020, the city had 16 districts under its jurisdiction, with a total area of 6,340.5 square kilometers. The built-up area covers an area of 1,237.85 square kilometers, with a permanent population of 24.2814 million and an urban population of 21.3919 million, with an urbanization rate of 88.10% (Shanghai Statistical Yearbook, 2020). In Shanghai, housing prices have been rising year after year, with the sales price of commercial housing rising from an average of 3,866 Yuan/m^2^ in 2001 to 23,804 Yuan/m^2^ in 2017 (China City Statistical Yearbook, 2001–2017). As one of the largest cities in China, the study of Shanghai can reflect the MWTP of people in the eastern and southern coastal areas of China. Thus, we choose Shanghai as the study area. In addition, to make the research more detailed, Shanghai is divided into 218 tracts based on the sixth National Population Census, as shown in Figure 1.

**Figure 2:**
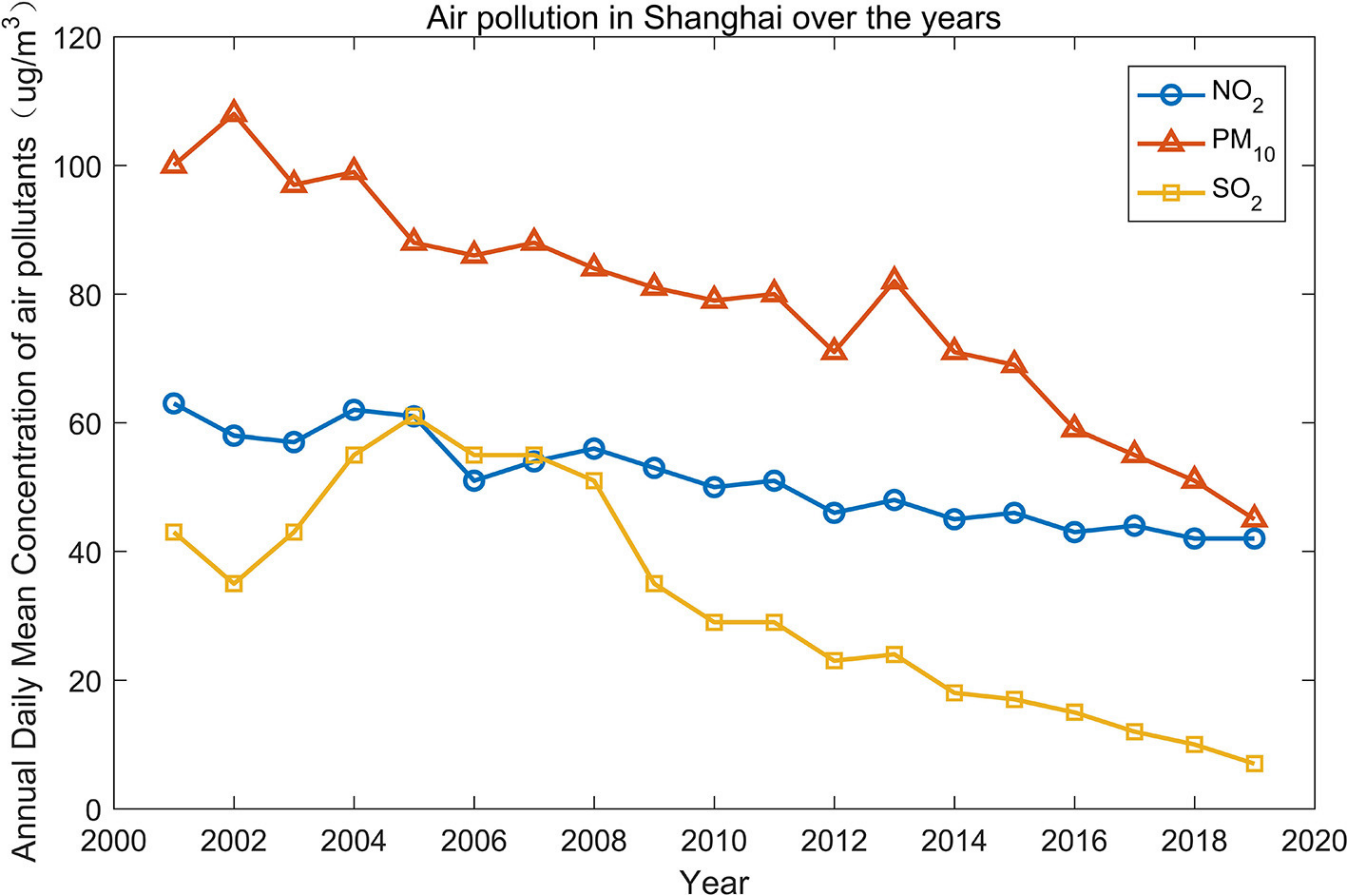
The annual mean concentrations of air pollutants in Shanghai, 2001–2019.

We focus on air pollutants around residential areas. Figure 2 shows the mean daily concentrations of SO_2_, NO_2_, and PM_10_ in Shanghai from 2001 to 2019. Overall, air quality in Shanghai has improved over the years, which is mainly due to the active governance of the Chinese government. The daily concentration of SO_2_ across different monitoring sites in Shanghai in 2019 was 7 μg/m^3^ (attaining the Grade I national standard for air quality), that of NO_2_ was 42 μg/m^3^ (exceeding the Grade II national standard of 2 μg/m^3^), and that of PM_10_ was 45 μg/m^3^ (attaining the Grade II national standard for air quality) (Shanghai Municipal Bulletin on the Status of Ecological Environment, 2019).

### Data Description and Processing

The data used in this study include housing price data, air quality data, and other built environment data in Shanghai. All the data used in this paper were collected in 2018 so that the time dimension can be unified.

The housing price information of 27,608 residential areas in Shanghai constituted the housing price data in this study. These data were taken from a platform for real estate transactions in Shanghai (http://cd.lianjia.com/) and included the address, average housing price, construction time, number of housing units, and latitude and longitude coordinates of each community.

The air quality data came from the Shanghai Municipal Bureau of Ecology and Environment. We collected data from 10 national controlled air quality monitoring stations of Shanghai in 2018. These data included the hourly mean concentrations of CO, O_3_, SO_2_, NO_2_, PM_10_, and PM_2.5_, the monitoring time, and the current air pollution levels, which can be used to calculate the annual mean concentrations of air pollutants in different census tracts of Shanghai.

**Figure 3:**
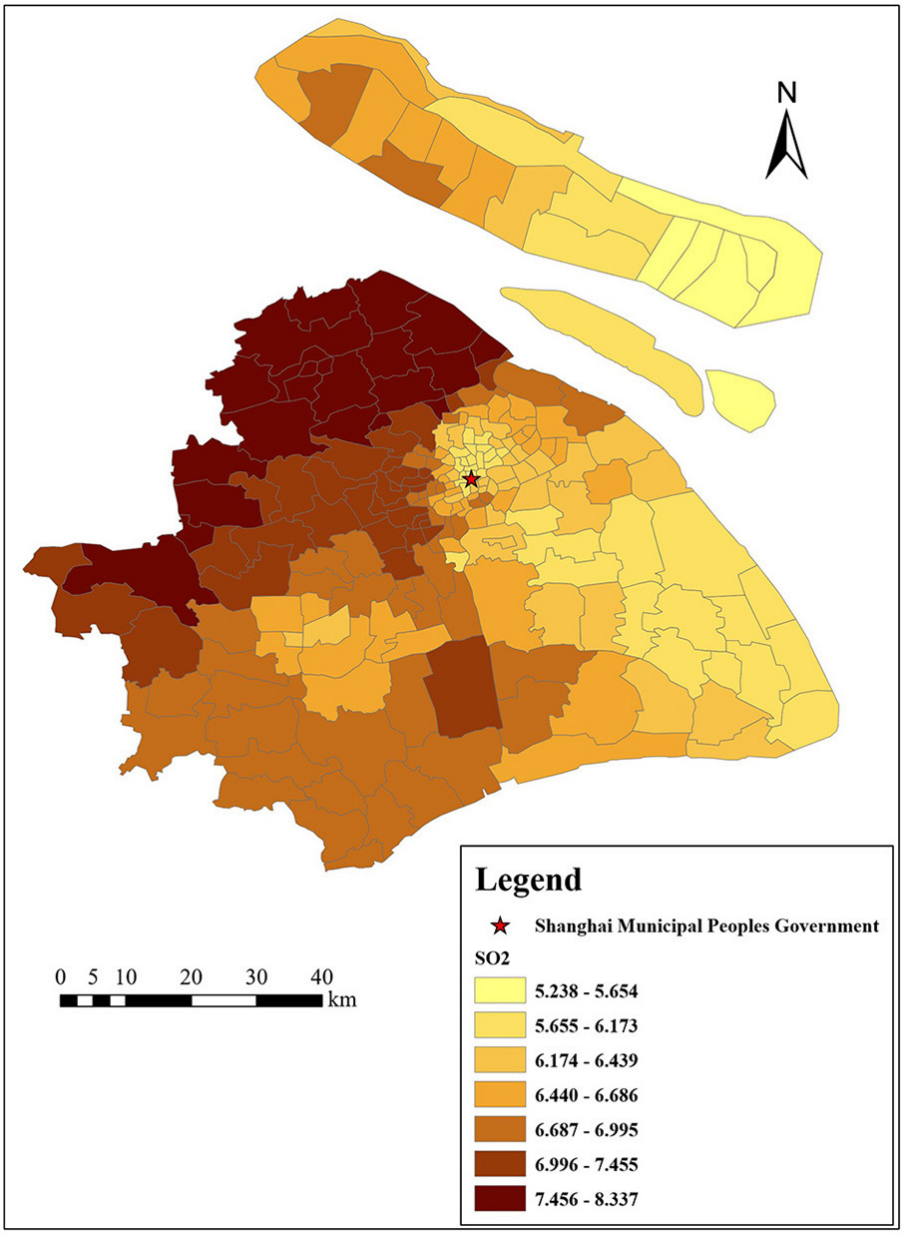
Distribution of SO_2_ (μg/m^3^) in Shanghai.

**Figure 4:**
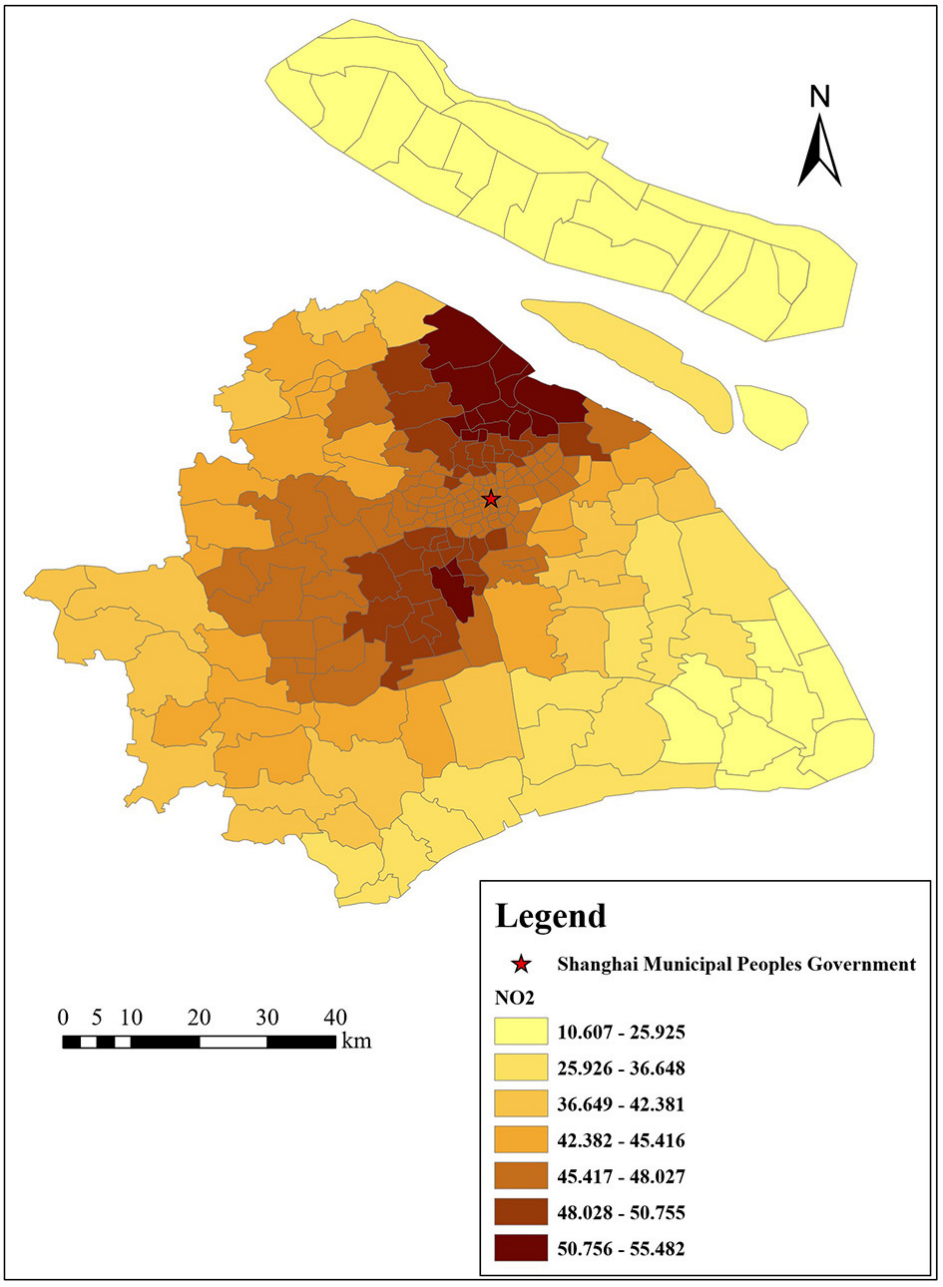
Distribution of NO_2_ (μg/m^3^) in Shanghai.

**Figure 5:**
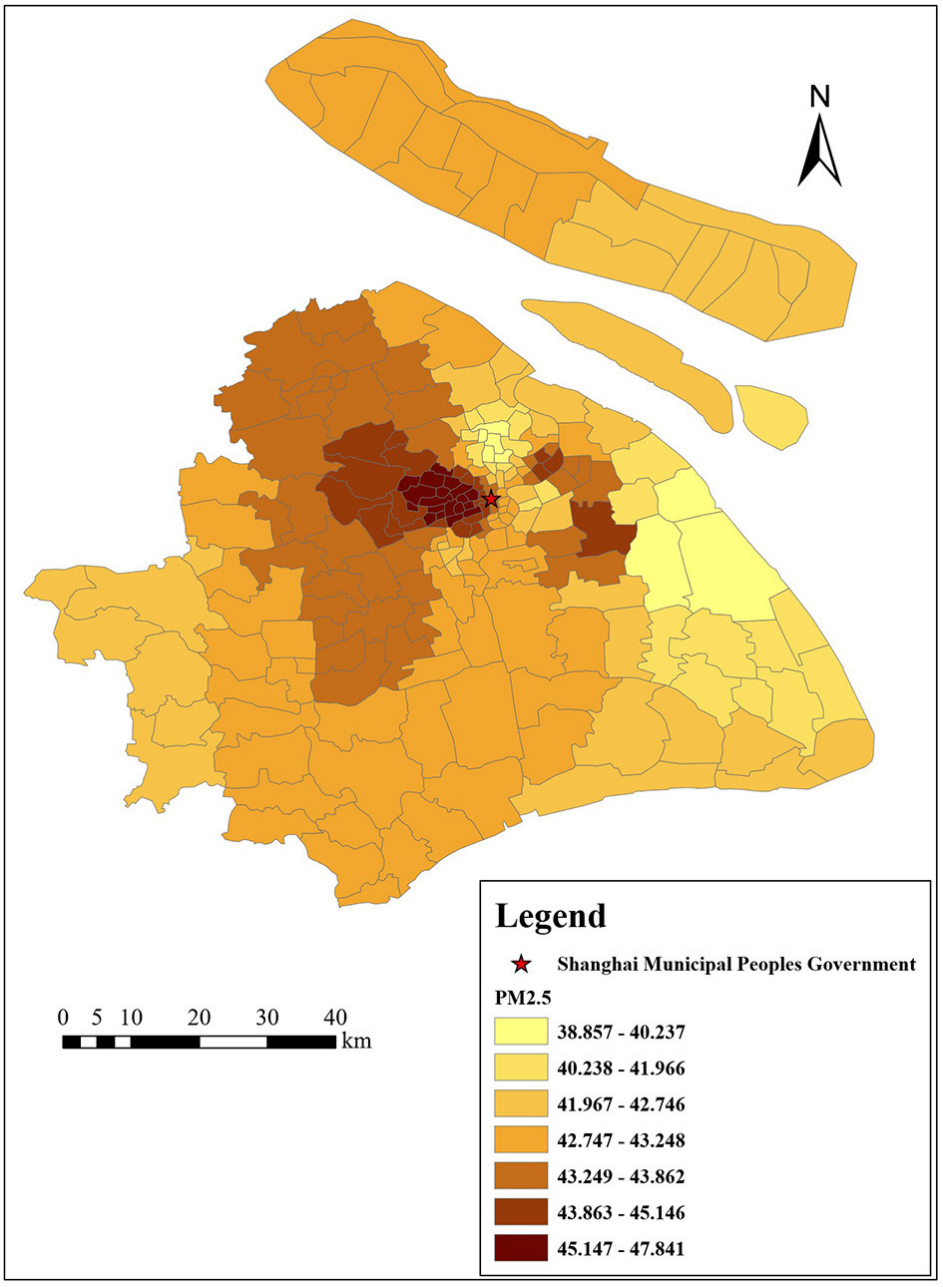
Distribution of PM_2.5_ (μg/m^3^) in Shanghai.

**Figure 6:**
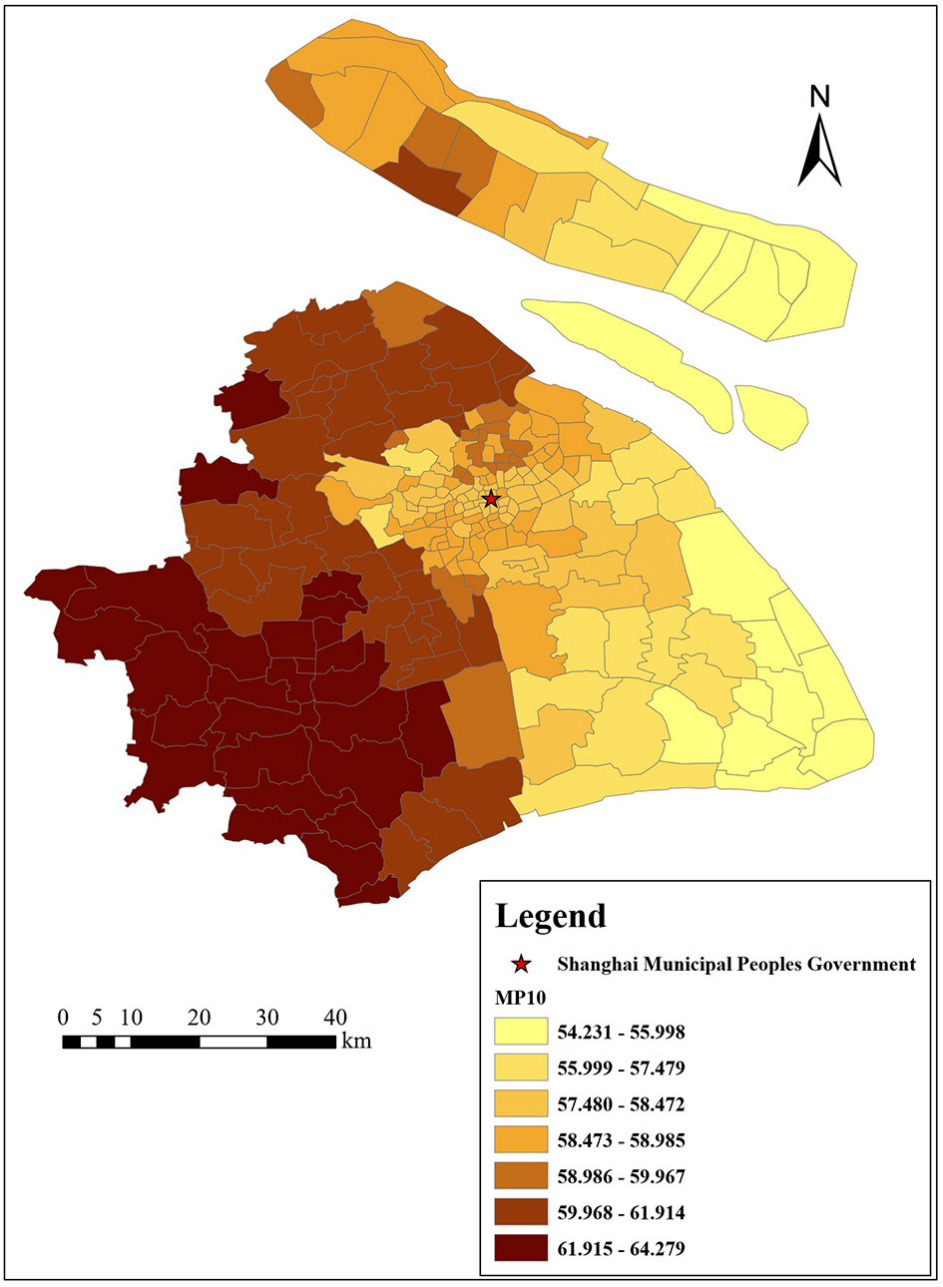
Distribution of PM_10_ (μg/m^3^) in Shanghai.

Figures 3–6 show that the air pollutants in Shanghai are diverse, mainly concentrated in the outer suburbs with heavy industries and the city center with heavy traffic. For example, the areas with the highest mean concentrations of SO_2_ and PM_10_ are the Baoshan District, Jinshan District, Yangpu District, and Jiading District, where there are many polluting factories, such as power plants, steel mills, and automobile factories. Furthermore, the large trucks that travel in these areas during the day also contribute to air pollution. The areas with the highest mean concentrations of NO_2_ and PM_2.5_ are mainly located in the inner ring area because of traffic congestion.

The built environment variables can be measured by the “5 Ds,” namely, density, design, diversity, distance to transit, and destination accessibility (42–44). In this study, the initial environmental data were composed of population data, urban road network data, and point of interest (POI) data. The population data came from the 6th Census of China. The urban road network data of Shanghai were extracted from the Open Street Map (OSM). The POI data were obtained from Baidu.com, which is one of the largest Chinese web mapping service applications. These data provided 14 types of locations, such as schools, restaurants, hospitals, parks, supermarkets, and transportation facilities (45). The total number of POIs in this dataset is 603,085, and for each POI, the basic information includes the name, type, location, and latitude and longitude.

**Figure 7:**
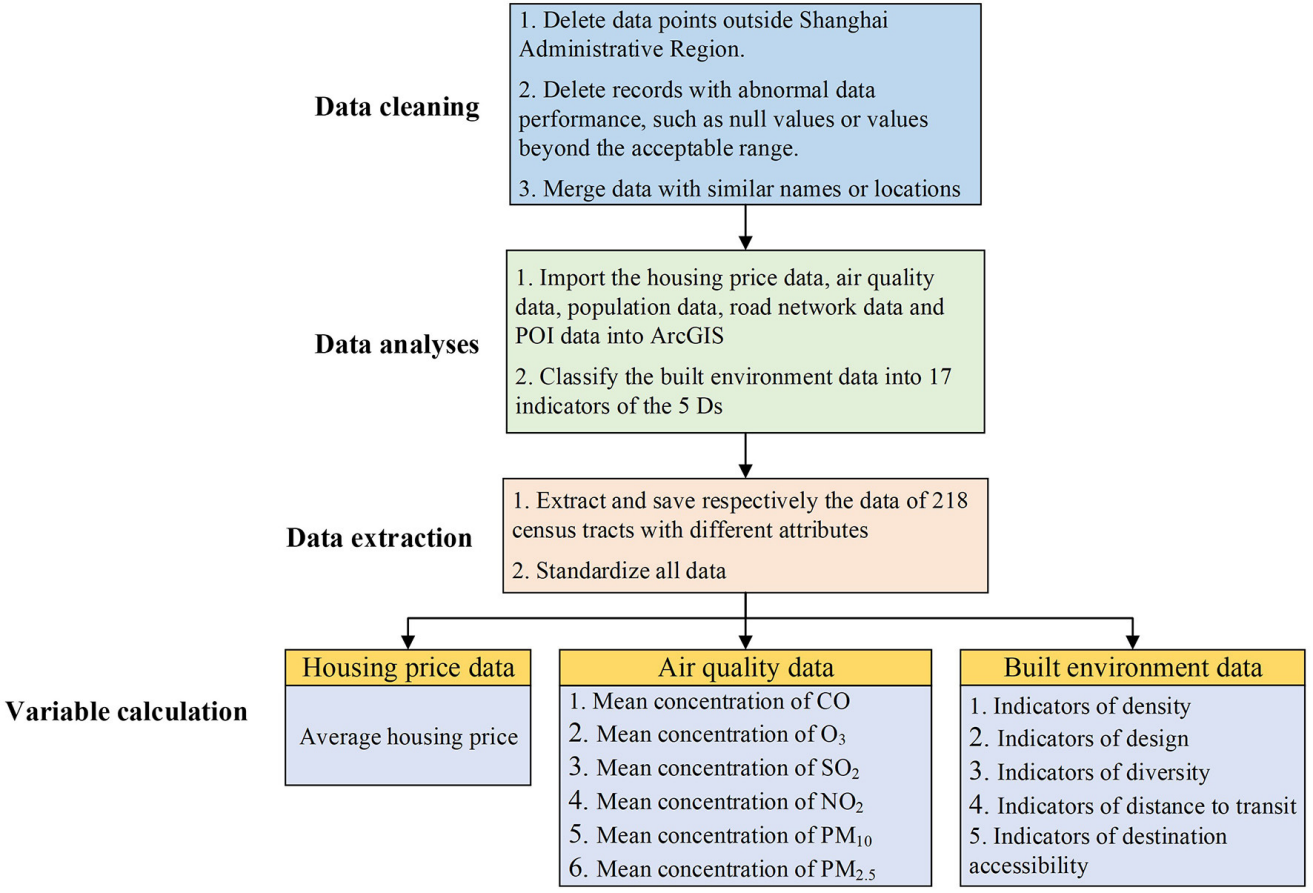
Diagram of the data processing.

The smallest research unit of this study is one of the 218 census tracts in Shanghai; thus, the three types of data above need to be processed. As shown in Figure 7, this processing mainly includes data cleaning, data analyses, data extraction, and variable calculation.

### Variable Calculation

Based on the existing data and referring to relevant references, we determined the dependent and independent variables of this study and performed a descriptive statistical analysis of all the variables, as shown in Table 2. There are 6 types of air quality data, namely, CO, O_3_, SO_2_, NO_2_, PM_10_, and PM_2.5_ data. The other built environment variables include 18 indicators of the “5 Ds.” In particular, land-use diversity is measured by the entropy index, which ranges in value between 0 and 1, where 0 means that the land use is single and homogeneous and 1 means that all types of land use are evenly distributed (43, 46). The calculation formula of land use diversity is defined as follows:


(1) $$\begin{array}{l} E_{i}=\underset{j}{\sum }\left(p_{ij}\ln\;p_{ij}\right)/\text{lnN} \end{array}$$

where *E*_*i*_ is the entropy for land uses within the spatial unit i, *p*_*ij*_ is the proportion of the *j*th land use at spatial unit i, and N is the number of land use categories. In this study, 14 land uses (restaurants, retail, hotels, tourist attractions, medical facilities, educational buildings, residences, parks, and so on) are considered (*N* = 14).

**Table 2:** Variable definitions and statistics.

**Variables**	**Variable description**	**Unit**	**Mean**	**S.D**.	**Min**	**Max**
**Dependent variable**						
Housing prices	Average housing price	Yuan^*^/m^2^	50,090	24,235	11,061	111,382
**Air quality variables**						
CO	Mean concentration of CO	μg/m^3^	0.59	0.04	0.45	0.71
O_3_	Mean concentration of O_3_	μg/m^3^	53.07	4.93	42.20	68.67
SO_2_	Mean concentration of SO_2_	μg/m^3^	6.67	0.55	5.23	8.34
NO_2_	Mean concentration of NO_2_	μg/m^3^	42.33	10.02	10.61	55.48
PM_10_	Mean concentration of PM_10_	μg/m^3^	59.19	2.09	54.23	64.28
PM_2.5_	Mean concentration of PM_2.5_	μg/m^3^	43.19	1.37	38.86	47.84
**Built environment variables**						
PopD	Population density	number/km^2^	15314	15971	68.06	68129
MetroSD	Metro station density	number/km^2^	0.30	0.40	0	1.96
MetroLD	Metro line density	km/km^2^	1.00	1.34	0	1.96
BusD	Bus stop density	number/km^2^	5.46	3.99	0.19	21.98
RoadD	Road network density	km/km^2^	8.96	6.24	0.34	39.11
PLD	Parking lot density	number/km^2^	29.52	34.78	0	150.25
Edu	Percentage of educational service POIs	%	0.32	0.47	0	3.63
Leis	Percentage of leisure place POIs	%	0.41	0.34	0.01	2.17
Stad	Percentage of stadium POIs	%	0.51	0.42	0	2.20
Med	Percentage of medical institution POIs	%	1.26	0.33	0	2.37
Park	Percentage of park POIs	%	0.77	0.44	0	3.36
Tour	Percentage of tourist attraction POIs	%	1.28	0.96	0	11.31
Super	Percentage of shopping mall and supermarket POIs	%	0.51	0.36	0.01	1.90
Tbh	Percentage of telecom business hall POIs	%	1.01	0.38	0	2.58
Res	Percentage of restaurant POIs	%	0.57	0.36	0	2.20
Landuse	Entropy index of the land use mix	–	0.23	0.17	0.06	0.95
Discent	The straight-line distance from the city center	km	19.78	15.70	0.18	58.64

* *A basic unit of the Chinese currency (RMB)*.

To measure the straight-line distance from the city center, we chose the location of the Shanghai Municipal People's Government as the downtown of Shanghai, where there are more than 10 large-scale shopping malls, the headquarters of dozens of well-known corporations, and hundreds of logistics, trade, information technology, and media enterprises.

In addition, the mean-centered method is used to eliminate the dimensional influence of all variables, including house prices. The formula is as follows:


(2) $$\begin{array}{l} x_{ik}=\frac{X_{ik}}{{\bar{X}}_{k}} \end{array}$$

where *x*_*ik*_ is the input value of the model, *X*_*ik*_ is the original value of the k-th characteristic variable at spatial unit i, ${\bar{X}}_{k}$ represents the original average value of the k-th variable.

## Methodology

Due to the obvious differences in the spatial distribution of air pollutants and housing prices (47, 48), we speculate that residents' MWTP for clean air is also spatially heterogeneous. Therefore, we choose a GWR model, which can represent the spatial variation in independent variables (49). In addition, to compare the applicability of the GWR model, we also establish the OLS model used in many studies (1, 29).

### Multicollinearity Test

Multicollinearity refers to the high linear correlation between explanatory variables, which makes a model difficult to estimate or the regression effect not ideal. To eliminate this phenomenon, we adopted the value of the variance inflation factor (VIF) to filter variables. The VIF is a measure of multicollinearity. Specifically, when the VIF value of a characteristic variable is >7.5, it has problems of multicollinearity with other independent variables and should be removed from the model (50). The VIF of the independent variables is positively associated with the coefficient of determination (*r*^2^) for the regression, which can be computed as follows:


(3) $$\begin{array}{l} VIF=\frac{1}{1-r^{2}} \end{array}$$

### Spatial Autocorrelation Test

Before using spatial regression models, the spatial autocorrelation of the variables should be tested. Moran's I test is often used to verify the spatial autocorrelation of variables (51), and Moran's I can be expressed as follows:


(4) $$\begin{array}{l} I=\frac{n}{\sum_{i=1}^{n}\sum_{j=1}^{n}w_{ij}}\cdot \frac{\sum_{i=1}^{n}\sum_{j=1}^{n}w_{ij}\;(x_{i}-\bar{x})\left(x_{j}-\bar{x}\right)}{\sum_{i=1}^{n}{\left(x_{i}-\bar{x}\right)}^{2}} \end{array}$$

where n is the number of spatial units, *w*_*ij*_ is the spatial weight between units i and j, *x*_*i*_ denotes the attribute value of unit i, and $\bar{x}$ represents the average value of all units.

Moran's I is a rational number, and it ranges between −1 and 1. Specifically, the larger the positive value of Moran's I, the stronger the spatial correlation. While the value of Moran's I <0 indicates a negative spatial correlation, and the smaller the value of Moran's I, the greater the spatial difference. A value near zero means a spatially random distribution. The null hypothesis of Moran's I test assumes that each explanatory variable is spatially independent, meaning that it is sufficiently close to 0. The Z-score is usually computed to verify the null hypothesis of Moran's I test and is defined as follows:


(5) $$\begin{array}{l} Z\;(I)=\frac{I-E\;(I)}{\sqrt{Var\;(I)}} \end{array}$$

where *E*(*I*) and *Var*(*I*) represent the expectation and standard deviation of Moran's I, respectively. The significance level in this study is *P* < 0.01, and the critical Z-score values are −2.58 and +2.58 when using a 99% confidence level.

### Geographically Weighted Regression Model

GWR models are based on the traditional linear regression model, which attempts to build a linear relationship between a given dependent variable and a set of independent variables (52). The GWR model tries to establish a linear regression equation for each spatial unit by considering the geographical changes among variables. As other observations are closer to the spatial unit, their influence on the coefficient estimation of this spatial unit is greater. This feature of the GWR model takes into account the local specific relationship between the dependent variable and independent variable of spatial variation during modeling. The calculation formula of the GWR model is as follows:


(6) $$\begin{array}{l} y_{i}=\beta_{0}\;(u_{i},v_{i})+\underset{k}{\sum }\beta_{k}\left(u_{i},v_{i}\right)\;x_{ik}+\varepsilon_{i}\;,\;i=1,2,3,\ldots ,n \end{array}$$

where *x*_*ik*_ is the independent variable, *y*_*i*_ is the dependent variable, (*u*_*i*_, *v*_*i*_) are the spatial latitude and longitude coordinate points of spatial unit i, ε_*i*_ is the Gaussian error term, $\varepsilon_{i}\sim N\;(0,\delta^{2}),Cov\;(\varepsilon_{i},\varepsilon_{j})=0\left(i\neq j\right)$, β_0_ (*u*_*i*_, *v*_*i*_) is the intercept of spatial unit *i* (the constant term), β_*k*_ (*u*_*i*_, *v*_*i*_) represents the relationship weight value of the k-th characteristic variable at spatial unit i, and n is the sample size of the spatial unit.

The implementation process of the GWR model is as follows:

**Step 1:** Determine the optimal bandwidth.

Bandwidth b is used to explain the functional relationship between *w*_*j*_ (*i*) (spatial weight function) and *d*_*ij*_ (the distance between spatial unit i and unit j). Excessive bandwidth will generate non-significant differences in parameter estimates between different regions, which will affect the accuracy of model parameter estimation. However, a bandwidth that is too small will lead to a large variation. The Akaike information criterion (AIC) is one of the criteria used to measure the optimal fitness of statistical models (53). In this study, the corrected AIC (AICc) is used to determine the optimal bandwidth. Compared with other methods of determining bandwidth, such as the cross-validation method and designated bandwidth method, the AICc method can usually obtain a better degree of fit. Specifically, the smaller AICc value indicates that the model is better. The calculation formula of AICc is defined as follows:


(7) $$\begin{array}{l} AICc\;\left(b\right)=2nln\;(\hat{\sigma })+2nln\;(2\pi )+n\left[\frac{n+tr\;(S)}{n-2-tr\;(S)}\right] \end{array}$$

where $\hat{\sigma }$ is the maximum likelihood estimate of the variance in the random error term, $\hat{\sigma }=\frac{RSS}{n}-tr\left(S\right)$, RSS denotes the sum of residual squares, and *tr*(*S*) is the trace of *S* matrix. The *S* matrix can be expressed as follows:


(8) $$\begin{array}{l} Ŷ=S\cdot Y \end{array}$$

where $Ŷ=\left[\begin{array}{c} \hat{y_{1}}\\ \begin{array}{c} \hat{y_{2}}\\ \begin{array}{c} \vdots \\ \hat{y_{n}} \end{array} \end{array} \end{array}\right]$. $Y=\left[\begin{array}{c} y_{1}\\ \begin{array}{c} y_{2}\\ \begin{array}{c} \vdots \\ y_{n} \end{array} \end{array} \end{array}\right]$.

Optimal bandwidth *b*_0_ corresponds to the minimum value of AICc, which is obtained as follows:


(9) $$\begin{array}{l} b_{0}=\underset{b>0}{\text{arcmin}}\;AICc\;(b) \end{array}$$

**Step 2:** Select the spatial weight function.

In general, spatial weight function *w*_*j*_ (*i*) is computed by the Gaussian kernel function or the Bi-square kernel function, which are both distance-decay functions. The weight of the Gaussian kernel is continuous and gradually decreases from the center of the kernel but never reaches zero, while the bi-square kernel has a specific range with a non-zero kernel weight, which controls the k-th nearest neighbor distance of each regression position. Furthermore, bandwidth b can be constant (fixed kernel) or variable (adaptive kernel). The adaptive kernel is suitable for creating a nuclear surface based on the density of sample points. If the distribution of elements is close, the coverage of the nuclear surface will be small; otherwise, it will be large.

In this study, we selected the adaptive Gaussian kernel function for the following two reasons. (1) We choose 218 census tracts of Shanghai with different areas, leading to denser tracts in the city center and fewer tracts in the suburbs. (2) Air quality is greatly affected by the distance factor. The adaptive Gaussian kernel function can be expressed separately as follows:


(10) $$\begin{array}{l} w_{j}\;(i)=\exp\left[-{\left(\frac{d_{ij}}{b}\right)}^{2}\right],\;j=1,2,3,\ldots ,n \end{array}$$

where *d*_*ij*_ is the distance between spatial unit i and unit j, which can be obtained by calculating the distance between the centroids of unit i and unit j by the Toolbox tool in ArcGIS; b is the optimal bandwidth, which can be obtained by Step 1.

**Step 3:** Compute the regression coefficient

Regression coefficient β_*k*_ (*u*_*i*_, *v*_*i*_) of spatial unit *i* can be obtained by the local weighted least square method. The calculation formula of parameter $\beta \;(i)={\left[\begin{array}{c} \beta_{0}\;(u_{i},v_{i})\;\beta_{1}\;(u_{i},v_{i})\;\ldots \;\beta_{k}\;(u_{i},v_{i}) \end{array}\right]}^{T}$ is as follows:


(11) $$\begin{array}{l} \beta \;(i)={\left[X^{T}W\;(i)\;X\right]}^{-1}X^{T}W\;(i)\;Y \end{array}$$

where $X=\left[\begin{array}{cc} 1 & \begin{array}{ccc} x_{11} & \ldots & x_{k1} \end{array}\\ \begin{array}{c} 1\\ \begin{array}{c} \vdots \\ 1 \end{array} \end{array} & \begin{array}{c} \begin{array}{ccc} x_{12} & \ldots & x_{k2} \end{array}\\ \begin{array}{ccc} \vdots & \ddots & \vdots \end{array}\\ \begin{array}{ccc} x_{1n} & \ldots & x_{kn} \end{array} \end{array} \end{array}\right]$, $W\left(i\right)=\left[\begin{array}{cccc} w_{1}\left(i\right) & \; & \; & \;\\ \; & w_{2}\left(i\right) & \; & \;\\ \; & \; & \ddots & \;\\ \; & \; & \; & w_{n}\left(i\right) \end{array}\right]=diag\;\left[\begin{array}{cccc} w_{1}\left(i\right) & w_{2}\left(i\right) & \ldots & w_{n}\left(i\right) \end{array}\right]$.

### Calculation of MWTP

MWTP represents the change speed of the current willingness to pay, that is, the coefficient of the independent variable (the slope), which means adding one unit of independent variables (such as NO_2_, PM_2.5_, the density of metro stations and road networks) and how much are residents willing to pay more for the housing price. However, to eliminate the dimensional influence of the independent variable and the dependent variable (the housing prices), the mean-centered method was adopted to normalize the variables in the GWR model. Therefore, the coefficients of the GWR model need to be transformed to calculate the MWTP. The specific formula is as follows:


(12) $$\begin{array}{l} {MWTP}_{ik}=\frac{\beta_{k}\;(u_{i},v_{i})\;\bar{Y}}{{\bar{X}}_{k}} \end{array}$$

where *MWTP*_*ik*_ is the MWTP of the k-th characteristic variable at spatial unit i, ${\bar{X}}_{k}$ represents the original average value of the k-th variable, and $\bar{Y}$ represents the average value of the dependent variable, which is the housing price in this paper.

## Results and Discussion

### Results Analysis

First, the Toolbox tool in ArcGIS was used to carry out multicollinearity tests on all the independent variables in Table 2, and the variables with VIF values >7.5 were removed. Then, stepwise OLS regressions were adopted to test the impact of independent variables on housing prices and select the variables with significant performance. Only 10 independent variables were left: the mean concentration of PM_2.5_, the mean concentration of NO_2_, metro station density, road network density, parking lot density, the percentage of educational service POIs, the percentage of stadium POIs, the percentage of medical institution POIs, the percentage of park POIs, and the straight-line distance from the city center.

The OLS regression results of these independent variables are shown in Table 3. The results show that PM_2.5_ and NO_2_ have a significant negative impact on housing prices, which are consistent with Chen and Chen (54) and Dong et al. (5), and the coefficients of other built environment variables are basically consistent with the theoretical and expected values. Specifically, Housing prices fall as the residential property move further away from the city center and housing prices rise with the increase of the number of subway stations, schools, and other infrastructures. Furthermore, the VIF values of these 10 independent variables are all relatively small, indicating that the collinearity among the variables is very weak.

**Table 3:** OLS regression results.

**Variables**	**Coefficient**	***t***	***P*-value**	**VIF**
Intercept	0.5995	10.7019	<0.000001***	/
NO_2_	−0.1263	−4.2164	<0.000001***	1.8592
PM_2.5_	−0.0769	−2.7490	0.0065***	1.6492
MetroSD	0.3144	5.0672	<0.000001***	2.7616
RoadD	0.7093	8.5758	<0.000001***	3.0392
PLD	0.1198	2.1490	0.0328**	2.1413
Edu	0.1510	1.9819	0.0489**	2.7115
Stad	0.2023	2.5412	0.0118**	5.2884
Med	0.1313	2.1091	0.0347**	3.4045
Park	0.2153	2.4739	0.0240**	1.9841
Discent	−0.5095	−12.8072	<0.000001***	3.2372

The ***p < 0.01 and **p < 0.05 statistical significance level, respectively.

The global Moran's I test was conducted to examine whether the 10 independent variables screened above had spatial autocorrelation. Table 4 shows the results, which include Moran's I, the expected index, the Z-score, and the *P*-value of the independent variables. All independent variables show spatial autocorrelation with a significance level of *P* < 0.01, and all Z-scores are >2.58. Therefore, it is necessary to use the GWR model to reveal the geographical variability of each variable.

**Table 4:** Results of Moran's I test.

**Variables**	**Moran's I**	**Expected index**	**Z-score**	***P*-value**
NO_2_	0.376775	−0.004831	31.485821	<0.000001
PM_2.5_	0.416511	−0.004831	34.653344	<0.000001
MetroSD	0.667202	−0.004831	55.290328	<0.000001
RoadD	0.756185	−0.004831	62.687319	<0.000001
PLD	0.494304	−0.004831	41.047587	<0.000001
Edu	0.611992	−0.004831	50.968685	<0.000001
Stad	0.814504	−0.004831	67.351090	<0.000001
Med	0.696556	−0.004831	58.052220	<0.000001
Park	0.464463	−0.004831	39.658548	<0.000001
Discent	0.727585	−0.004831	60.034994	<0.000001

**Figure 8:**
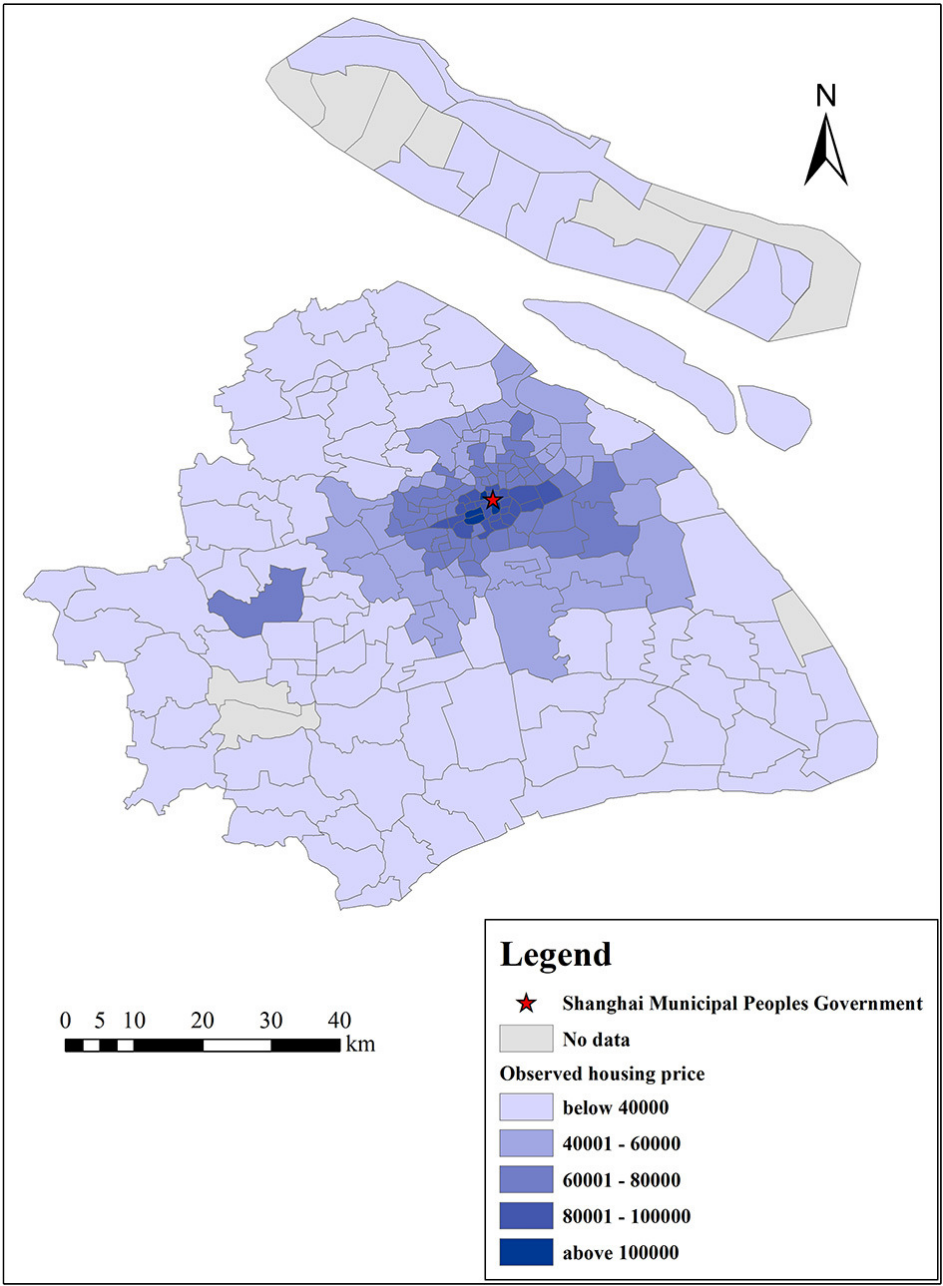
Observed housing prices (Yuan/m^2^) in Shanghai.

**Figure 9:**
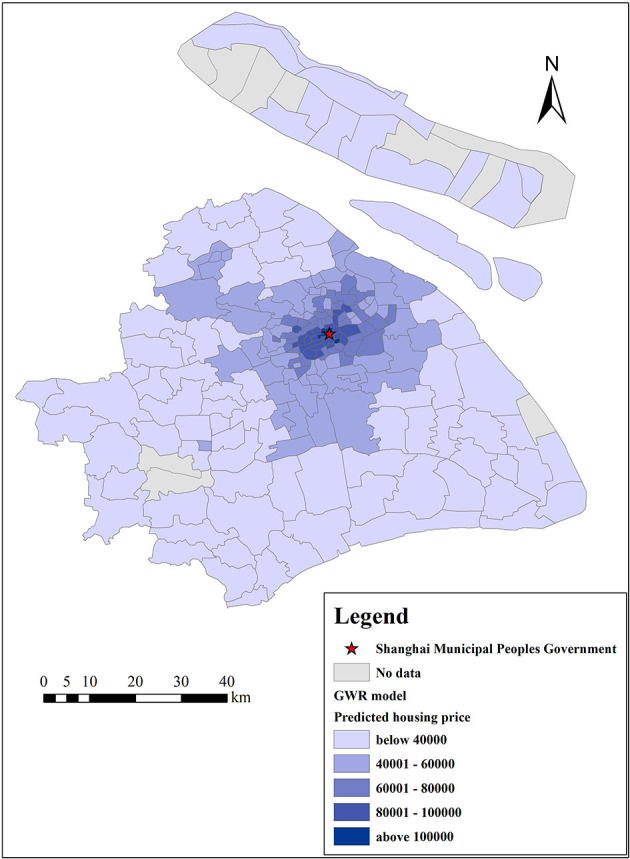
Predicted housing prices (Yuan/m^2^) in Shanghai based on the GWR model.

Figures 8, 9 show the actual housing prices and the housing prices predicted by the GWR model, indicating that the GWR model has a high degree of fitting accuracy. To further prove the superiority and feasibility of the GWR model, we compared it with the OLS model, and both models adopted the same explanatory variables mentioned above. We computed the root mean square error (RMSE), mean absolute error (MAE), mean relative error (MRE), *R*^2^, adjusted *R*^2^, and AICc values of these two models, as shown in Table 5. From these six indicators, we can find that the prediction accuracy of the GWR model is better than that of the traditional OLS model. Meanwhile, the MAE and *R*^2^ of the GWR model are 5,029 and 0.876 Yuan/m^2^, respectively, which further reflects the high prediction accuracy of the GWR model. Furthermore, we computed 6 eigenvalues of the estimated coefficients of different variables to describe their influence range, namely, the average value, minimum value, maximum value, lower quartile, median, and upper quartile, as shown in Table 6. From the average value of the estimated coefficient, the GWR model results are consistent with those of the OLS regression as a whole, which shows that the mean concentration of PM_2.5_, mean concentration of NO_2_, and distance from the city center have negative effects on housing prices and that the other independent variables are all positively correlated. In addition, the value symbols of the maximum and minimum values are the same, proving that there is no directional difference in the influence of different variables on housing prices in Shanghai, and all variables are either promoting or inhibiting factors.

**Table 5:** OLS and GWR comparison results.

**Indicators**	**OLS**	**GWR**
RMSE (Yuan/m^2^)	10,746	6,981
MAE (Yuan/m^2^)	8,499	5,029
MRE	0.233	0.187
*R* ^2^	0.803	0.876
Adjusted *R*^2^	0.793	0.863
AICc	−313.374	−396.005

**Table 6:** GWR modeling results (the coefficients of different variables).

**Variables**	**Average**	**Minimum**	**Maximum**	**Lower quartile**	**Median**	**Upper quartile**
Intercept	0.4621	0.2258	0.7611	0.3692	0.4590	0.5418
NO_2_	−0.0838	−0.2114	−0.0086	−0.1388	−0.0626	−0.0269
PM_2.5_	−0.0430	−0.1407	0.0140	−0.0700	−0.0350	−0.0085
MetroSD	0.2962	0.2822	0.3218	0.2868	0.2922	0.3050
RoadD	0.6182	0.5609	0.7381	0.5743	0.6006	0.6572
PLD	0.0856	0.0443	0.1953	0.0641	0.0738	0.0975
Edu	0.1412	0.1363	0.1516	0.1383	0.1401	0.1435
Stad	0.2004	0.1566	0.2476	0.1880	0.2001	0.2138
Med	0.1671	0.1053	0.1957	0.1527	0.1759	0.1857
Park	0.2028	0.1791	0.2534	0.1937	0.1983	0.2072
Discent	−0.6203	−1.5254	−0.0896	−0.8114	−0.5871	−0.3664

### Spatial Heterogeneity Analysis of MWTP

**Figure 10:**
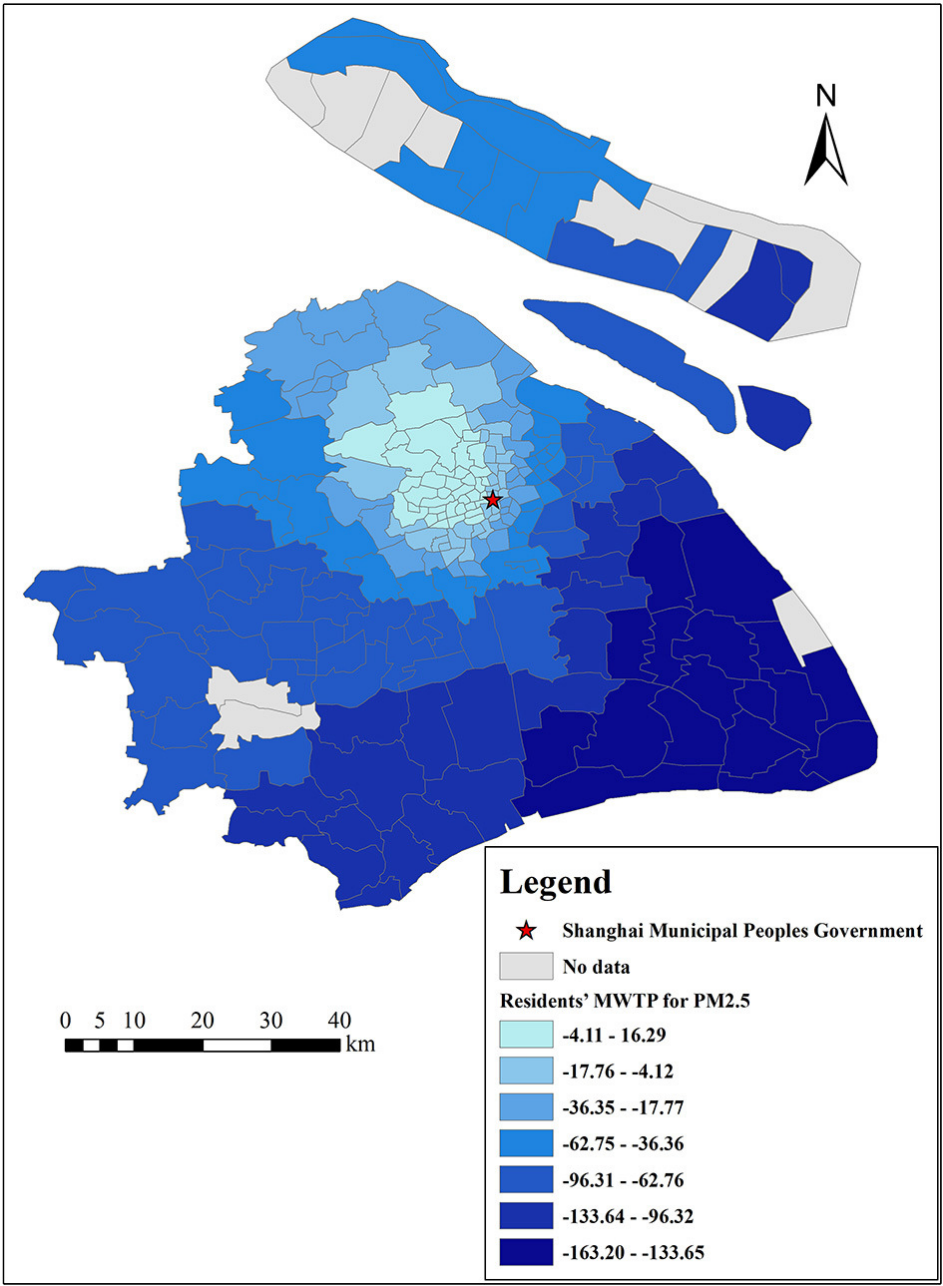
The spatial heterogeneity of MWTP for PM_2.5_.

**Figure 11:**
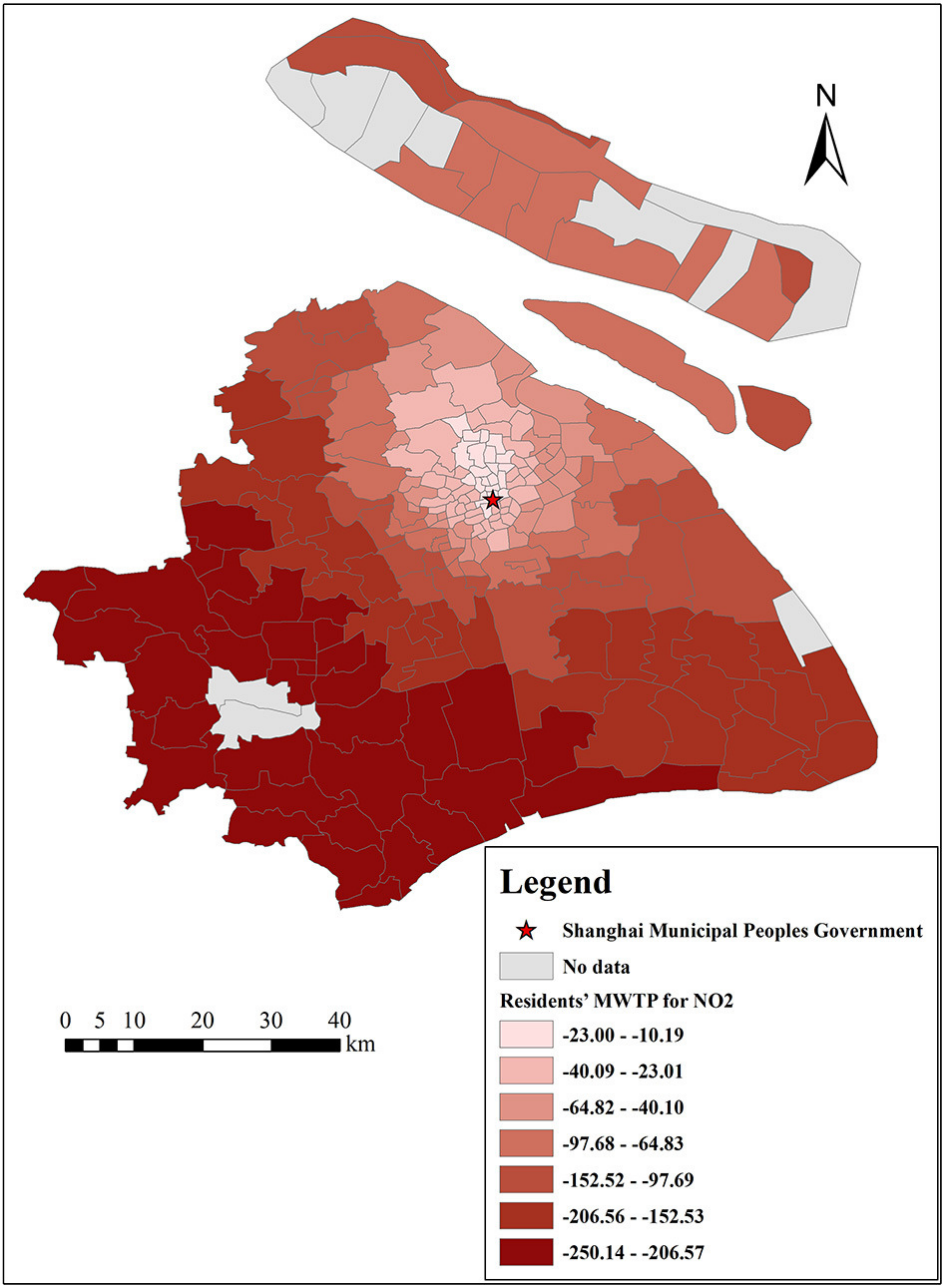
The spatial heterogeneity of MWTP for NO_2_.

**Figure 12:**
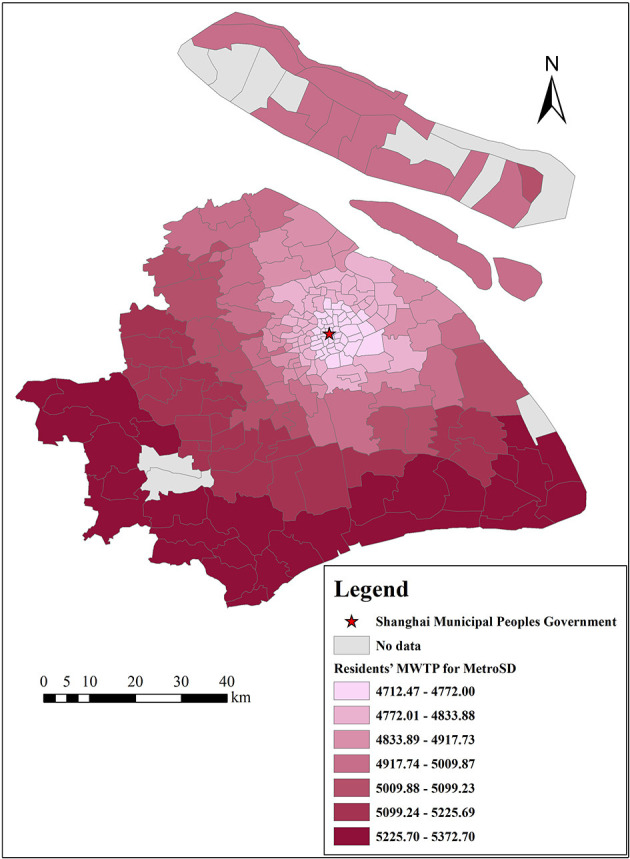
The spatial heterogeneity of MWTP for metro station density.

**Figure 13:**
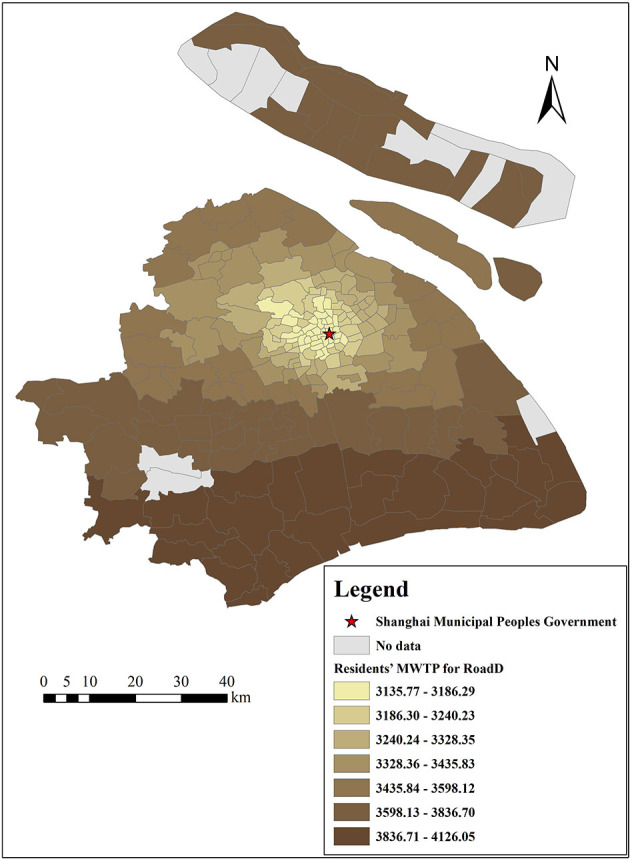
The spatial heterogeneity of MWTP for road network density.

**Figure 14:**
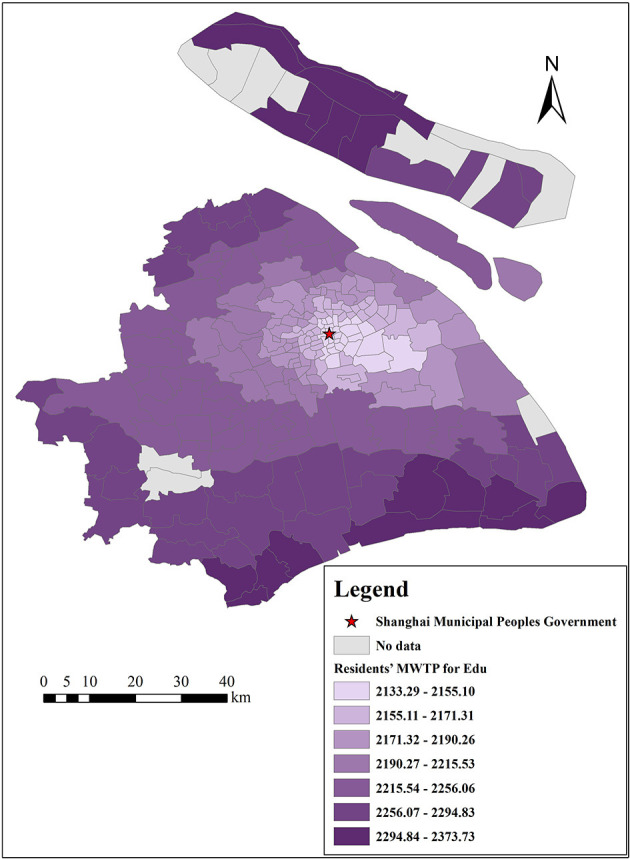
The spatial heterogeneity of MWTP for educational services.

**Figure 15:**
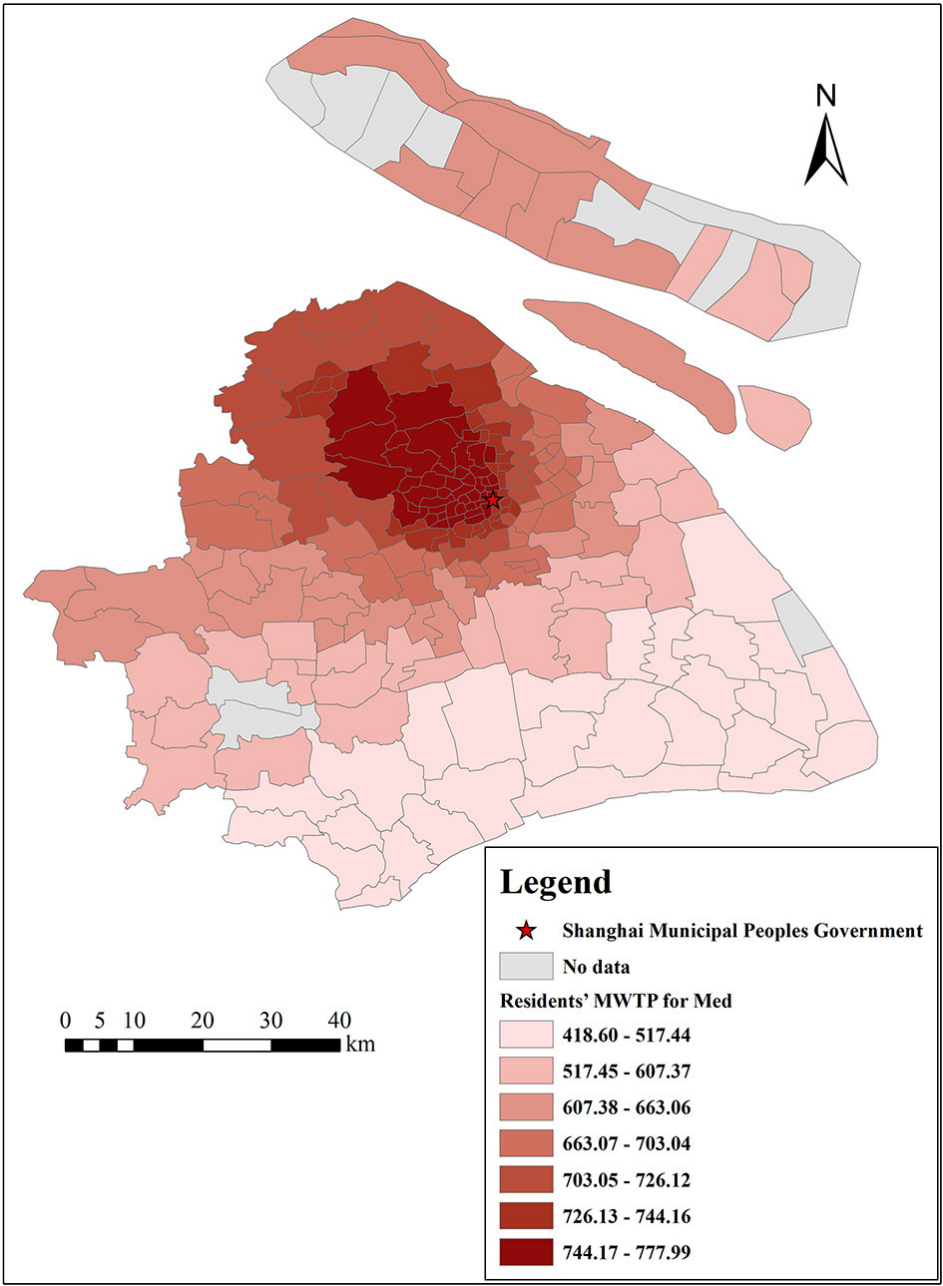
The spatial heterogeneity of MWTP for medical institutions.

**Figure 16:**
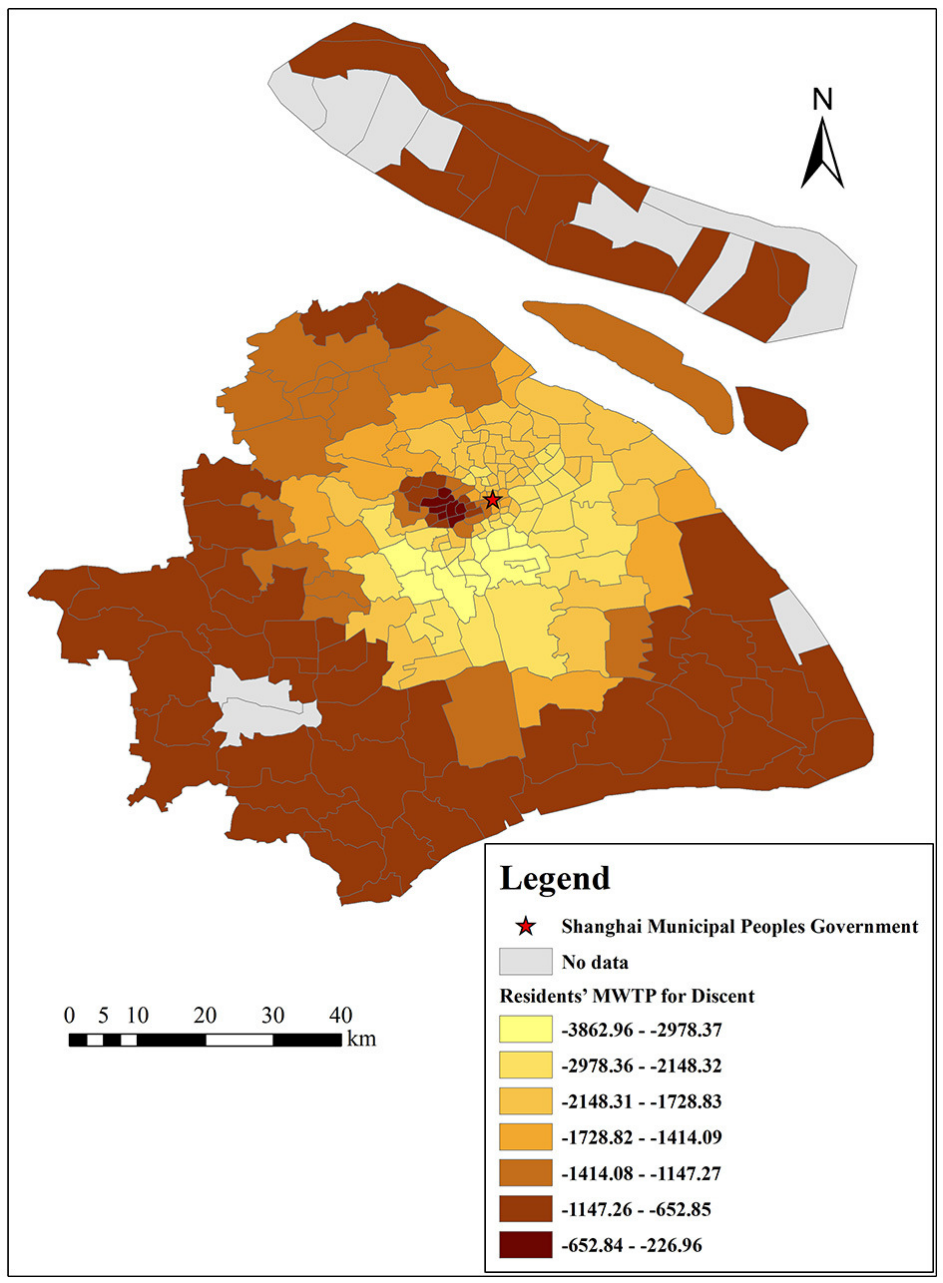
The spatial heterogeneity of MWTP for the distance from the city center.

There are many estimates of coefficients output by the GWR model because one important characteristic of GWR models is that the estimated coefficients of each independent variable vary with each census tract, which indicates that each selected variable is affected by local characteristics (49). Therefore, the results of the GWR model can provide a reference for the government to formulate regional policies. In addition, we can calculate residents' MWTP for each variable by Equation (12). This study focuses on the analysis of residents' MWTP for clean air, as shown in Figures 10, 11, and the MWTP for other variables are shown in Figures 12–**16**.

Figures 10, 11 indicate that residents' MWTP for clean air has obvious spatial heterogeneity, Shanghai residents, on average, are willing to pay 50 Yuan/m^2^ to reduce the mean concentration of PM_2.5_ by 1 μg/m^3^, and the lowest absolute value of MWTP is 16 Yuan/m^2^ in the central Jing'an District and the highest absolute value of MWTP is 163 Yuan/m^2^ in the Pudong New Area. Shanghai residents are, on average, willing to pay 99 Yuan/m^2^ to reduce the mean concentration of NO_2_ by 1 μg/m^3^. The lowest absolute value appears in the Putuo District, where it is 10 Yuan/m^2^, while the highest absolute value appears in the Qingpu District, where it is 250 Yuan/m^2^. In other words, ceteris paribus, our results show that a 1 μg/m^3^ reduction in the mean concentrations of PM_2.5_ and NO_2_ results in a 0.1 and 0.2% increase in Shanghai property value, respectively (in 2018, the average housing price in Shanghai was 50,090 Yuan/m^2^, and the mean concentrations of PM_2.5_ and NO_2_ were 43.19 and 42.33 μg/m^3^, respectively). Meanwhile, this result is slightly lower than that in developed countries, such as the US (1 μg/m^3^ reduction in TSPs increases the value of housing by 0.2–0.4%) (9), and it is similar to the study of prefecture level cities in China (a 1 μg/m^3^ increase in the PM_2.5_ is associated with up to a 36 Yuan/m^2^ reduction in housing prices) (19, 20). We speculate that there are two possible reasons: (1) China is currently a developing country, and people pay less attention to air pollution than developed countries. (2) Housing prices in Shanghai are very high, resulting in a small proportion of clean air.

This result shows that Shanghai residents' MWTP for reducing NO_2_ levels is higher than that for reducing PM_2.5_ levels, which is similar to the OLS model results. The reason is that the effect of NO_2_ on Shanghai residents is more obvious. NO_2_ is the main pollutant that causes acid rain, and the acid rain frequency was 53.8% in 2018, up 6.2% from the previous year (Shanghai Municipal Bulletin on the Status of Ecological Environment, 2018). Moreover, NO_2_ can cause photochemical pollution and swelling of the human lungs (55). Furthermore, residents' MWTP for reducing levels of PM_2.5_ and NO_2_ increased from the city center to the suburbs. The possible reasons are as follows: (1) There are many factories in the suburbs that emit a lot of air pollutants, especially thermal power plants, machinery manufacturing industry, and automobile factories (Shanghai Municipal Bulletin on the Status of Ecological Environment, 2019), such as heavy machinery manufacturing in Pudong New Area, Shanghai Volkswagen in Jiading District, and petrochemical industry in Jinshan District (Shanghai Statistical Yearbook, 2020). Compared with traffic emissions, air pollutants emitted by these factories are concentrated and visible. Therefore, suburban residents living near these factories may be more disgusted with the air pollution and more willing to pay for clean air (56, 57). (2) Even though there are fewer infrastructure resources in the suburbs, more and more people move from the urban areas to the suburbs in order to enjoy a better quality of life, and these people attach great importance to the living environment (Shanghai Municipal People's Government, 2021). However, residents who live near the city center value other built environmental factors, such as convenient transportation, closer workplaces, and abundant educational and medical resources (1, 5, 24). (3) The result may be different from some previous studies on multiple cities (from a macro perspective) or multiple communities (from a micro perspective) with large economic differences (5, 9, 31). Because our study focuses on each census tract in Shanghai (from a meso perspective), where the economic gaps are relatively small. Even some rich people may prefer to move to the suburbs to enjoy better quality of life, especially in the five new towns.

The density of metro stations and road networks are both important indicators of urban traffic convenience. Figures 12, 13 show the spatial variation in residents' MWTP for the density of metro stations and road networks, respectively, with estimated MWTP ranging from 4,712 to 5,373 Yuan/m^2^ and from 3,136 to 4,126 Yuan/m^2^, respectively. Both indicators are positively correlated with housing prices. In addition, there is a decreasing trend from the suburbs to the city center of residents' MWTP for metro stations and road networks. The possible reasons are as follows: the density of metro stations in the center of Shanghai is relatively dense, with 1 subway station per km on average and 1 subway station per 5–10 km in the suburbs (Shanghai Statistical Yearbook, 2020). The density of road networks in the center of Shanghai is also relatively large, with a dense distribution of secondary trunk roads and branch roads, while it is sparse in the suburbs. Therefore, suburban residents are more eager to increase the density of metro stations and road networks. This result suggests that compared to increasing the density of metro stations and road networks in the city center, increasing these two indicators in the suburbs is more effective for improving the real value of land, especially in areas with poor traffic services.

Figures 14, 15 show the spatial differences in Shanghai residents' MWTP for educational services and medical institutions, with estimated MWTP ranging from 2,133 to 2,377 Yuan/m^2^ and from 419 to 778 Yuan/m^2^, respectively. In general, educational services and medical institutions are positively correlated with housing prices. Spatially, residents' MWTP for educational services is lower in the city center and higher in the suburbs, while it is opposite for residents' MWTP for medical institutions. The reason is probably that China advocates “going to school nearby,” and Chinese residents attach great importance to school district houses, while there are fewer schools in the suburbs, and suburban residents are crazier about school district houses, even though such houses are more expensive. Regarding medical institutions, people are eager to obtain the best medical services when they are sick, and the city center has abundant medical resources. Therefore, both urban and suburban residents are more willing to go to the city center to see a doctor, so residents' MWTP for medical institutions is higher in the city center. For these reasons, it is necessary to promote the balanced development of education and medical care between the city center and the suburbs of Shanghai.

Figure 16 shows the spatial impact of the straight-line distance from the city center on housing prices. In general, the distance from the city center is negatively correlated with housing prices, and the range of the GWR model's coefficient is from −0.0896 to −1.5254. Based on Equation (12), we convert it to MWTP, which ranges from −227 to −3,863 Yuan/m^2^. Spatially, the absolute value of MWTP for the distance from the city center is higher in the inner suburbs, while it is lower in the downtown and outer suburbs. The reason is probably that residents in the inner suburbs are more dependent on downtown resources (such as shopping malls, parks, and stadiums), which will make them tend to buy houses as close to the city center as possible. This result indicates that residents in the inner suburbs are more sensitive to the distance from the city center, while residents in the outer suburbs are more sensitive to other built environmental factors of housing, such as air quality and traffic convenience.

### Actual Losses Caused by Air Pollution in Shanghai

Based on the above estimates, we attempt to compute the actual losses caused by air pollution in Shanghai from the perspective of asset value depreciation in the entirety of the Shanghai housing market. In 2018, the total building area of the housing stock in Shanghai was 594.6 million m^2^ (Shanghai Academy of Social Sciences, 2019). If the mean concentrations of PM_2.5_ and NO_2_ increased by 1 μg/m^3^, the estimated losses of the entire Shanghai real estate market would be 29.730 and 58.865 billion Yuan, respectively.

In general, our research proves that air pollution has caused great economic losses in Shanghai. Shanghai residents are very sensitive to air pollution, and residents' MWTP for clean air is lower in the city center and higher in the suburbs, especially in the outer suburbs, such as the Qingpu District, Pudong New Area, and Jinshan District.

## Conclusions and Implications

Under the poor air quality conditions in China, residents' demand for clean air increases as their incomes rise (58). To improve public health and formulate regionally specific air quality improvement plans and policies, we adopted a GWR model to explore the spatial heterogeneity of residents' MWTP for clean air in different areas of Shanghai. The main findings are as follows:

(1) In this case study, the GWR model performed better than the OLS model for the same variables, with significantly smaller AICc values, higher *R*^2^ values, and adjusted *R*^2^ values.

(2) At the level of Shanghai as a whole, air pollutants have a negative impact on housing prices. Using the marginal rate of transformation between housing prices and air pollutants, Shanghai residents, on average, are willing to pay 50 and 99 Yuan/m^2^ to reduce the mean concentration of PM_2.5_ and NO_2_ by 1 μg/m^3^, respectively.

(3) From the perspective of Shanghai census tracts, residents' MWTP for clean air has obvious spatial heterogeneity. Suburban residents pay more attention to air quality, which indicates that they have a higher MWTP for clean air, especially in the southeast, such as Pudong New Area, and southwest, such as Jinshan District and Songjiang District. In contrast, residents in the city center value other built environment factors, such as convenient traffic and abundant educational resources.

(4) We also measured the actual losses caused by air pollution in Shanghai from the perspective of housing market value. The results show that if the mean concentrations of PM_2.5_ and NO_2_ increased by 1 μg/m^3^, the estimated losses of the entire Shanghai real estate market would be 29.730 and 58.865 billion Yuan, respectively.

The view that air quality can be capitalized by housing prices is fully confirmed in this study. Based on the conclusions above, several policy recommendations are proposed:

(1) Air pollutants seriously endanger people's health and can lead to cancer, respiratory diseases, and low immunity of humans (55, 59). The intensification of air pollution will also lead to an increase in the cost paid by residents. The measurement of MWTP can evaluate the value of air quality, a public good that is difficult to be priced through the market mechanism. This study can provide a scientific and empirical basis for evaluating the economic benefits of environmental protection projects and environmental governance policies. Relevant government departments can conduct a preliminary benefit-cost analysis of pollution control.

(2) NO_2_ can cause photochemical pollution and swelling of the human lungs (60). Compared with PM_2.5_, Shanghai residents have higher MWTP for reducing NO_2_, which is mainly related to congested traffic and the boiler waste gas. The government should promote the development of the new energy vehicle industry and encourage citizens to take public transportation or carpool to go out. City policymakers should design scientific and reasonable air pollution control measures and improve the relevant regulations and policy system of atmospheric environment control.

(3) Regionally targeted air governance policies contribute to the rapid improvements in public health and land value. City policymakers can use differential taxation and government intervention to improve the energy structure and reduce industrial emissions. The areas with higher MWTP for clean air should be considered as key areas for air pollution control, such as Jinshan District, Pudong New Area, and Jiading District. Boiler retrofitting, urban sprinkling and dust suppression can be strengthened by charging air taxes and enterprise sewage tax.

(4) Air quality control requires a lot of funds, and the source of funds is the practical difficulties faced by local governments. The results of MWTP can provide a possible reference for the financing of related public health governance projects (61). In policy practice, the government can adopt a value slightly lower than Equation (12) based on housing price to levy environmental tax, which can be used to increase investment in environmental renovation on the one hand and improve the overall welfare of homebuyers on the other hand.

This study computes residents' MWTP for clean air in different census tracts of Shanghai, and it can help Shanghai and other cities achieve a win-win situation of economic development and public health. However, this study has some limitations. In this study, the GWR model is linear and cannot reflect the non-linear relationship between the independent variables and housing prices. In the future, we can develop non-linear machine learning algorithms based on spatial and temporal dimensions. Furthermore, more variables affecting housing prices (per capita income, the demographic structure, land policy, etc.) should be considered in the model development of future studies.

## Data Availability Statement

The original contributions presented in the study are included in the article/supplementary material, further inquiries can be directed to the corresponding author/s.

## Author Contributions

ZL: data curation, validation, original draft preparation, and software. XL: validation, data curation, writing, reviewing, and editing. WL: conceptualization and methodology. YL: supervision. GZ: visualization. MT: methodology and editing. All authors contributed to the article and approved the submitted version.

## Funding

This study was supported by the National Natural Science Foundation of China (Grant Nos. 71961137006 and 52002244), Shanghai Pujiang Program (Grant No. 2020PJC083), and Shanghai Chenguang Program (Grant No. 20CG55).

## Conflict of Interest

The authors declare that the research was conducted in the absence of any commercial or financial relationships that could be construed as a potential conflict of interest.

## Publisher's Note

All claims expressed in this article are solely those of the authors and do not necessarily represent those of their affiliated organizations, or those of the publisher, the editors and the reviewers. Any product that may be evaluated in this article, or claim that may be made by its manufacturer, is not guaranteed or endorsed by the publisher.
