# Corrigendum: Exploring the Spatial Heterogeneity of Residents' Marginal Willingness to Pay for Clean Air in Shanghai

**DOI:** 10.3389/fpubh.2022.854259

**Published:** 2022-02-10

**Authors:** Ziliang Lai, Xinghua Liu, Wenxiang Li, Ye Li, Guojian Zou, Meiting Tu

**Affiliations:** ^1^The Key Laboratory of Road and Traffic Engineering, Ministry of Education, Shanghai, China; ^2^College of Transportation Engineering, Tongji University, Shanghai, China; ^3^Department of Traffic Engineering, Business School, University of Shanghai for Science and Technology, Shanghai, China; ^4^University of Paris-Saclay, Automatic, School of Information and Communication Technologies, Paris, France

**Keywords:** air pollution, housing prices, geographically weighted regression, marginal willingness to pay, spatial heterogeneity

There is an error in the Funding statement. The grant numbers for the Funding source “The National Nature Science Foundation of China was incorrectly written as “Grant Nos. 7181101155 and 52002244.” The “Grant No. 7181101155” in the original article is actually an application number rather than a grant number. The correct number for “National Natural Science Foundation of China” is “Grant Nos. 71961137006 and 52002244.”

The authors apologize for this error and state that this does not change the scientific conclusions of the article in any way. The original article has been updated.

## Publisher's Note

All claims expressed in this article are solely those of the authors and do not necessarily represent those of their affiliated organizations, or those of the publisher, the editors and the reviewers. Any product that may be evaluated in this article, or claim that may be made by its manufacturer, is not guaranteed or endorsed by the publisher.

